# Recommendations on complementary feeding for healthy, full-term infants

**DOI:** 10.1186/s13052-015-0143-5

**Published:** 2015-04-28

**Authors:** Patrizia Alvisi, Sandra Brusa, Stefano Alboresi, Sergio Amarri, Paolo Bottau, Giovanni Cavagni, Barbara Corradini, Linda Landi, Leonardo Loroni, Miris Marani, Irene M Osti, Carlotta Povesi-Dascola, Carlo Caffarelli, Luca Valeriani, Carlo Agostoni

**Affiliations:** Department of Paediatrics, Ospedale Maggiore, Azienda USL, Bologna, Italy; Department of Paediatrics and Neonatology, Ospedale di Imola, Azienda USL, Imola, Italy; Outpatient Paediatrician, Azienda USL, Bologna, Italy; Department of Paediatrics, Arcispedale S. Maria Nuova, Azienda USL-IRCCS, Reggio Emilia, Italy; Coordinator European Allergology Center – European Diagnostic Center Dalla Rosa Prati, Parma, Italy; Department of Dietetics and Clinical Nutrition, Ospedale Maggiore-Bellaria, Azienda USL, Bologna, Italy; Paediatrician, Ospedale privato Accreditato S. Francesco, Ravenna, Italy; Freelance paediatrician, Cesena, FC Italy; Clinica Pediatrica, Department of Clinical and Experimental Medicine, University of Parma, Parma, Italy; Department of Clinical and Community Sciences, University of Milano, IRCCS Fondazione Cà Granda, Ospedale Maggiore Policlinico, Milano, Italy

**Keywords:** Complementary feeding, Allergy, Coeliac disease, Diabetes, Metabolic syndrome, Nutrition requirements, Baby led weaning

## Abstract

Weaning (or introduction of complementary feeding) is a special and important moment in the growth of a child, both for the family and the infant itself, and it can play a major role in the child’s future health. Throughout the years, various weaning modes have come in succession, the latest being baby-led weaning; the timing for introducing foods and the requirements of which sort of nutrient for weaning have also changed over time. Furthermore, the role played by nutrition, especially in the early stages of life, for the onset of later non-communicable disorders, such as diabetes, obesity or coeliac disease has also been increasingly highlighted.

Members of Italian Society of Gastroenterology, Hepathology and Pediatric Nutrition (SIGENP) and the Italian Society of Allergology and Pediatric Immunology (SIAIP) Emilia Romagna here propose a practical approach for pediatricians to deal with daily practice. The four main areas for discussion were weaning in relation with the onset of allergic diseases, coeliac disease, diabetes and metabolic syndrome, the nutrition requirements to take into account for assessing the diet of infants under one year of age and about the practice of baby-led weaning focusing on limits and benefits, respectively.

## Introduction

Weaning or the introduction of complementary feeding is an important moment in the growth of a child, both for the family and the infant itself, and it can play a major role in a child’s future health.

Different weaning practices have characterized this stage of life according to traditions, ethnical origins and scientific beliefs. As a matter of fact, the typical schedules indicating the *correct* times of solid introduction were quite rigid, thus negatively influencing the infant’s natural attitude towards a progressive adjustment to a new diet.

This document has been jointly drafted by members of Italian Society of Gastroenterology, Hepathology and Pediatric Nutrition (SIGENP) and the Italian Society of Allergology and Pediatric Immunology (SIAIP) Emilia Romagna, working both in hospital and outpatient clinics, with the support of nutrition experts from the Italian Society for Parentheral and Entheral Nutrition (SINPE) and the European Society of Pediatric Gastroenterology, Hepatology and Nutrition (ESPGHAN). Its aim is, starting from data and information drawn from the literature on the topic, to provide suggestions on weaning modes, trying to “de-medicalize” this natural stage of an infant’s life.

*Weaning is the period of time when infants introduce food different from milk in their diet, together with a gradual reduction of the intake of milk (either breast milk or formula), to finally and gradually acquire their family’s diet model.*

This definition is based on the one proposed by the European Food Safety Authority (EFSA) [1] and by the European Society for Pediatric Gastroenterology, Hepatology and Nutrition (ESPGHAN) [2].

The beginning and the gradual completion of weaning are the result of a balanced number of factors that allow infants to nourish themselves in an increasingly autonomous and complete way [3]:

- ✓ acquisition of fundamental milestones in neuromotor development
- ✓ development of taste and personal inclinations
- ✓ maturation of renal and gastrointestinal functionality [4,5]
- ✓ qualitative and quantitative implementation of nutritional intake
- ✓ interaction of cultural and socioeconomic factors with local and family traditions [1]

A recent European multicenter study (5 countries involved: Belgium, Germany, Italy, Poland, Spain) studied, among other issues, the average weaning age, thus partly picturing habits in our continent in this respect [6]. Data show that, overall, about 25% of children start weaning before 4 months of age and, at 6 months of age, at least 90% has already eaten solid food. The study also shows that weaning age is lower for formula-fed children as compared to breast-fed children. At 4 months, 37.2% of formula-fed infants has started weaning, versus a percentage of 17.2% for breast-fed children; at 6 months of age, these percentages rise to 96.2% and 87.1% respectively.

*The stage of introduction of solid food, according to international literature on the topic* [1,2]*, is between 17 and 26 weeks of age (4–6 months) when the 6 months limit is not possible. The choice of the right moment to start weaning shall have to be determined not only by nutritional needs, but also by assessing the child’s “neurological maturity” and interest for food* [1,2]*. As for exclusively breast-fed infants, EFSA suggests that:*

*“[…] Exclusive breast-feeding provides adequate nutrition up to 6 months of age for the majority of infants, while some infants may need complementary foods before 6 months […] in addition to breast-feeding in order to support optimal growth and development”.*

In this document, we will analyze the relation between the introduction of complementary feeding and the impact it might have on the child’s health. More specifically, we will discuss what role the introduction of foods different from milk might play in relation with allergic diseases (part I), type 1 diabetes mellitus, coeliac disease and metabolic syndrome (part II) and give indications on infant nutritional requirements and some practical suggestions (part III).

## Part I: Complementary feeding and allergy

A progressive increase in the frequency of allergic disease and food allergy, especially among children in the western world has been found [7-9]. In 2007, a meta-analysis [10] has observed that self-reported prevalence of food allergy varied from 1.2% to 17% for milk, 0.2% to 7% for egg, 0% to 2% for peanuts and fish, 0% to 10% for shellfish, and 3% to 35% for any food. More recently, Nwaru et al. [11] assessed papers from 2000 to 2012 and observed a pooled lifetime and point prevalence of self-reported food allergy were 17.3% (95% CI: 17.0–17.6) and 5.9% (95% CI: 5.7–6.1), respectively while the point prevalence of sensitization to #1 food as assessed by specific IgE was 10.1% (95% CI: 9.4–10.8) and by skin prick test 2.7% (95% CI: 2.4–3.0), and by food challenge positivity 0.9% (95% CI: 0.8–1.1). The causes for this increasing are not completely clear yet: it is likely that this data might be the result of a combination between genetic predisposition, environmental factors, changes in lifestyle and nutrition habits, especially diet in the first months of life [12].

Based on these data and on some studies published in the ’90s, some prestigious scientific societies such as the American Academy of Pediatrics (AAP) [13], the American College of Asthma Allergy and Immunology [14] and the European Academy of Allergy and Clinical Immunology (EAACI) [15], had issued recommendations on weaning for children at risk for allergy, which included a late introduction of allergenic foods, such as milk after 12 months of age and egg after 24 months. These recommendations were based on a supposed “immaturity” in the mucosal immunity of infants, which was supposed to favor a sensitization towards alimentary antigens [16].

On the contrary, recent studies on animal models suggested that tolerance to food might be regulated/guided by an early and regular exposition to food proteins during a “critical window” which would open at 4 months of age and close at 6 months [17]. In support of this, recent observational studies highlights that a delay of the introduction of foods does not reduce the frequency of allergic disease and sensitization and might even increase the risk of atopy [18-20]. A cohort study showed that the introduction of cereal before 5 and a half months of age, fish before 9 months and egg before 11 months, as compared to later introductions, reduces the risk of asthma, allergic rhinitis and atopic sensitization at 5 years of age [20]. However, interventional studies have provided discordant results. On the one hand the early introduction of peanut decreases sensitization and occurrence of allergy to peanut [21], and on the other hand this does not happen for the egg [22]. Overall, these data suggest that a late introduction of foods (after 6 months of age) in an infant’s diet is not useful to prevent allergies. Along this line, in the latest suggestions issued by AAP [12], EAACI [23], ESPGHAN [2] e EFSA [1] it is recognized that there is no scientific evidence to justify the delayed introduction of solid foods, even those recognized as more allergenic, in order to prevent allergic diseases.

At the moment, there is no scientific evidence in support of the promotion of a deliberately early exposure (before 4 months of age) to the main allergenic foods [11]. Against this hypothesis , it has been prospectively shown that the introduction of solid food after 17 weeks of age is associated with a lower risk of food allergy [24].

Further important data emerging from recent literature point at a favoring role of breast milk for the development of tolerance; thus, weaning while continuing breastfeeding might reduce the onset of allergies [3,19,24]. So, it is highly recommended to introduce solid foods during breastfeeding, as breast milk is an ideal food for an infant’s nutrition. We therefore, believe it would be reasonable to avoid, if possible, a “strict” timetable for introducing new foods, and to follow the infant’s tastes.

### Key points

- When it is possible, an infant should be breast-fed during the stage of introduction of solid foods.
- There is no evidence that a delayed introduction of solid foods after 6 months of age, both in children at risk of atopy (with parents or siblings suffering from allergic diseases) and in those who are not at risk, might prevent allergy.
- The role played by the introduction of solid foods between 17 and 26 weeks of age in favoring the development of tolerance is still uncertain.

## Part II: Complementary feeding and coeliac disease, type 1 diabetes mellitus, metabolic syndrome

### Coeliac disease (CD) and type 1 diabetes mellitus (T1DM)

Previous observational studies seemed to suggest that gluten introduction between 4 and 6 months of age could reduce the risk of coeliac disease [25]. More specifically, ESPGHAN 2008 recommendations suggested to avoid both early introduction (<17 weeks) and late introduction (>26 weeks) of gluten, trying to use the window of tolerance indicated by American studies [2]. It was also recommended to gradually introduce gluten while the infant was still being breast-fed.

### Role of breastfeeding

Some studies seem to find a protective role of breast-milk for CD [26] and a weak protective effect for T1DM [27]. For CD it is not clear whether it is persistent protection or delay in the onset of symptoms. No study, as a matter of fact, shows long-term protective effects or effects depending on the duration of breastfeeding [25,28]. For T1DM, the protective effect might be linked with the duration and exclusiveness of breastfeeding, but there is no consensus in the scientific community about it [27,29].

### Timing of gluten introduction

Data from observational studies suggest that an early introduction (before 4 months of age) and above all a late introduction (after 6 months of age) of gluten might increase the risk of CD [25,30]. New recent data from randomized trials do not confirm the possible benefit related either to the timing of exposure or the introduction of small amounts of gluten. More specifically, the CELIPREV study showed how late introduction does not reduce the risk of CD. Two groups of children at risk for family history received gluten respectively at 6 and 12 months of age. At 5 years of age, 16% of children of both groups had received a diagnosis of CD. No further difference was detected at 10 years of follow up. The only significant difference was the timing of the onset of the disease: the group with an earlier timing of gluten introduction had an earlier diagnosis (26 vs 34 months) [31].

On the other hand there’s not consensus on the risk of developing T1DM in relation with the age of gluten introduction [32,33]. In the follow up of the BABYDIET study, the introduction of gluten at 6 months was compared to an introduction at 12 months and no difference was found for the incidence of diabetes mellitus [34].

### Amount of gluten

The results of a Swedish observational epidemiological study point out that the administration of gluten in large amounts would favor the development of CD as compared to the administration of small-medium amounts. Infants gradually introduced between 4 and 6 months, preferably while still breastfed, had a significantly lower prevalence of CD as compared to counterparts that consumed gluten in high amounts ever since its introduction [25]. A recent randomized study, the Prevent CD study, showed no advantage in introducing small amounts of gluten at 16 weeks of age. Infants at high risk of CD for family history and genetics, divided into two groups, received from 16 to 24 weeks of age, 100/mg/day of gluten or placebo respectively. At 3 years of age, the incidence of CD in the two groups was comparable: 5.9% in group 1 and 4.5% in group 2. (P = .47). The duration of breastfeeding did not modify the incidence of CD either [35].

### Early introduction of other foods and T1DM

The early introduction (in the first 4 months) of cow milk, fruit and fruit juice also seems to indicate an increased risk of developing T1DM autoantibodies, but research results in this respect are few and fragmented [36].

In conclusion, recent literature failed to confirm that introducing gluten between week 17 and 26 while the infant is still being breast-fed would have a protective role on the onset of CD, T1DM and wheat allergy. A protective role of breast feeding for CD and T1DM has not been proven. Similarly, the timing of gluten introduction does not seem to have an effect on the subsequent development of CD and T1DM. In any case, breastfeeding should be also promoted for its unique role in establishing maternal-infant bonding.

### Metabolic syndrome

The term *Metabolic Syndrome* (MS) refers to a clinical condition at increased cardiovascular risk due to the presence of multiple factors, such as visceral obesity, dyslipidemia, a state of insulin resistance and arterial hypertension. Although some observational studies suggested that an early introduction of complementary food might increase the risk of overweight/obesity, with a lower risk for breast-fed as opposed to formula-fed infants [37], there is no evidence that the age of introduction of complementary foods has an effect on the risk of developing obesity [38], type 2 diabetes, coronary disease and hypertension [39].

The data available in literature suggest that, between 6 and 24 months of age, a protein intake of more than 15% of total energy can lead, in some subjects, to early adiposity rebound phenomena, thus favoring the development of future obesity. Accordingly, an excess protein would stimulate the secretion of insulin and IGF1, responsible both for adipogenesis and the differentiation of adipocytes [40]. Nevertheless, the relation between protein intake during weaning and later risk of hypertension and cardiovascular disease, is still unclear [41]. Energy remains in any case a main determinant for fat deposition. In general, substituting hypercaloric and high-protein foods with foods having a lower energy density (cereal, fruit and vegetables) may be a possible approach to reduce the risk of obesity [42].

No association has been found between a high intake of fats with weaning and obesity in the following ages; on the contrary, Rolland Cachera identified in a hyperproteic and hypolipidic pattern of infants living in developed countries a possible contributing factor of early adiposity rebound [43]. Therefore, together with a reduction of proteins, an increase in the percentage of fats in the diet should be considered as an important step in the prevention of a condition of overweight [44].

Fruit juices (100% fruit), fruit drinks, vegetable juices and other sweetened beverages (*soft drinks, sweetened water with or without aromas, sweetened instant tea*) are defined as EPL (Energy Providing Liquids). There is no nutritional benefit in administering EPLs to infants in their first months of life: an excessive consumption of sweetened beverages and the consequent increase in caloric intake is associated with childhood obesity [45]. AAP suggests that infants < 6 months of age should not drink fruit juices and for infants beginning weaning, until one year of age, whole, pureed or homogenized fruit is recommended.

An early intake of salt might lead to a preference for salty tastes, with a consequent persistently high intake of salt also in the following ages, which can lead to increased arterial pressure [46].

There is no extensive literature on the role of LCPUFA (long chain n-3 polyunsaturated fatty acids) during weaning and their long-term effects on cardiovascular health. A Danish study showed that healthy infants receiving fish-oil supplements presented a lower arterial pressure, confirming the results yielded by a previous follow-up study carried out by Forsyth et al. [47,48].

### Key points

- The timing introduction and the amount of gluten does not seem to have an effect on the following development of CD and T1DM. Gluten can be introduced at any age after 6 months. While a protective role of breastfeeding for CD and T1DM has not been proven, breastfeeding should be supported as well.
- There is no clear correlation between the timing of introduction of complementary feeding and the risk of obesity, type 2 diabetes and cardiovascular disease in later ages.
- An excess in energy intake is still a primary factor leading to overweight*.
- Excessive protein intake (milk, meat, cheese) more than 15% total calories during weaning has been found to correlate with the risk of overweight/obesity in later age, in parallel with a decrease in fat percentage.
- An excessive consumption of sweetened beverages before 1 year of age and a consequent increase in caloric intake is associated with obesity in childhood.
- It is inappropriate, during the first year of age, to add salt to food, due to a possible increase in the risk of hypertension in later age.
- The introduction of LCPUFA from fish can have a positive effect on arterial pressure in the following ages.
*we recommend the use of WHO growth charts for breast-fed infants* [49].

## PART III: Requirements: energy, macronutrients, fibers, water, micronutrients and vitamins

International recommendations provide specific indications on the need and suitability of exclusive breastfeeding for the first 6 months of an infant’s life [1]. Nutritional supply, in fact, including that of micronutrients, is guaranteed by breast-milk, with the exception of vitamin D and K. In the second half of the first year of life, breast-milk alone is not enough to supply a sufficient amount of calories, proteins, zinc, iron and fat-soluble vitamins (vitamins A, D, K), necessary to guarantee an adequate growth to the infant [1,2].

The recommended intakes suggested below for infants between 6 and 12 months of age are extracted from the LARN (Reference Levels of Assumption of nutrients and energy for the Italian population) document, reviewed in 2012 [50] and from the EFSA (European Food Safety Authority) 2013 document [51]. Such requirements should only serve as a suggestion for the pediatrician, in case it was necessary to carry out an assessment of every single nutritional intake.

### Energy

#### The daily intake of energy in terms of total calories recommended is 70–75 Kcal/Kg/day

Such intake should be correctly distributed among the various macronutrients, both in terms of quantity and quality. It is extremely important to respect such daily energy intake, as the current trend is that of exceeding in the calories introduced daily.

### Macronutrients and fibers

*Carbohydrates: the recommended daily intake varies between 45% and 60% of total calories*.

It is advisable to prefer starchy alimentary sources, if possible with a low glycemic index and, above all, it is strongly recommended to reduce the intake of simple sugars (such as fruit juices, sugar and sweeteners in general). More specifically, it would be better to prefer simple starchy foods, especially bread, pasta, rice, other minor cereals (corn, oat, barley, spelt, etc.) as well as potatoes.

*Lipids: recommended intake is 40% of total calories, and should not be less than 25%*.

Qualitatively, the consumption of saturated fats, which are found mainly in foods of animal origin, should be limited (<10% of total energy) , while unsaturated, polyunsaturated and monounsaturated fats, found mainly in foods of vegetable and fish origin, should be preferred. Long-chain polyunsaturated fatty acids (LCPUFA), contained in vegetable oils (extra virgin olive oil) for fatty acids of the ω-6 series, in some kinds of fish (mackerel, anchovies, salmon, tuna, etc.) and fish oils for fatty acids of the ω-3 series, need to be introduced through diet with a recommended intake of 250 mg/day.

*Proteins: the daily recommended intake is about 10% of total calories; the recommended intake is 1.1 g/Kg/day (average recommended intake 11 g/day for 6 months of age)*.

Recommended protein intake has been reduced in the past few years, as hyperproteic diets seem to favor the onset of obesity (see paragraph 2.3).

According to ESPGHAN, it would be advisable not to introduce cow milk as the main source of milk before 12 months of age, as it is poor in iron and seems to possibly cause intestinal microhaemorrhages [2]. However, this recommendation is not confirmed elsewhere and has no unanimous scientific foundations. Furthermore, we believe it might pose problems for many families who cannot afford formula milk. From a practical standpoint, it may depend on the daily dose of milk the infant and the young child are accustomed to.

Tables 1 and 2 report protein intake of some kinds of meat and fish and of baby foods move here the original Tables 2 and 3.

**Table 1:** **Protein content of some kinds of meat and fish**

***Kind of meat/fish***	***g proteins/portion of 20–30 g***
Guineafowl, breast	5.2-7.7
Turkey, breast	4.8-7.2
Chicken, breast	4.7-7
Pork, steak	4.3-6.4
Veal/steer/beef fillet	4.1-6.2
Whole chicken (without skin)	3.9-5.8
Rabbit/horse	4-6
Cod/sole	3.4-5.1

**Table 2:** **Protein content of some baby foods**

***Food***	***Amount***	***Grams of protein***
Lyophilized beef	100 gr/10 g	50/5
Homogenized beef	120 g/100 g/80 g	7.4/6.2/5
Fresh fish	100 g	19
Fresh legumes	100 g	7
Dry legumes	100 g	24
Type 2 formula milk	100 ml	1.4-1.5
Cow milk	100 ml	3.3

**Table 3:** **Fiber content of some vegetables**

***Low fiber content***	***Medium fiber content***	***High fiber content***
Potato	Carrot	Peas
Chard	Zucchini	Green beans
Lettuce	Spinach	

Fibers: the recommended daily intake is not precisely quantified in children of 6–12 months. From 1 to 3 years the adequate intake is 10 gr/day [52].

An adequate intake of legumes, fruit and vegetables is also recommended.

Correct with Table 3 reports the fiber content of some vegetables.

A constant, but still moderate, intake of dietary fibers can be obtained by encouraging the consumption of cereals and derivatives, also in their unrefined form (pasta, rice, bread, breakfast cereal, etc.).

*Liquids: the daily recommended intake of liquids is 100 ml/Kg*.

It is advisable to consume water and avoid other drinks, which often present a high content of sugars.

### Vitamins and mineral nutrients

*Vitamin D: the recommended daily intake is 400 IU*.

Only 10% of this requirement is introduced through diet. 90% is synthesized through the effect of UVB rays on the precursor found on the skin.

Foods containing vitamin D are cod liver oil and fat fishes, butter and fat cheeses and eggs.

*Calcium: a daily intake of 260 mg is recommended.*

Foods containing calcium are milk and its derivatives (in children younger than 1 year they contribute for 65% of the daily intake), vegetables (12% of daily intake), cereal (8.5% of the daily intake), meat and fish (6.5% of total). Type 2 formula milk contains 230 mg of calcium /100 ml, follow-on formula 70 mg/100 ml, while cow milk 120 mg/100 ml.

*Sodium: a daily intake of 0.4 g is recommended.*

The evidence of a direct correlation between sodium consumption and arterial hypertension is uncertain in children.

It is advisable not to add salt to food until 1 year of age, as the sodium content of some foods used in the early stages of is enough to cover the daily requirement.

*Iron: a daily intake of 7 mg is recommended.*

Infants on exclusive breastfeeding who do not introduce complementary feeding are at risk of developing an iron deficit in the second semester.

Foods containing iron are meat and fish (bovine: 1.9 mg/100 g; cod 0.7 mg/100 g) and some vegetables (legumes, endive, green chicory, spinach). Absorption, however, is different, as the iron found in fish and meat is absorbed for about 25%, while the percentage of iron absorbed from vegetables varies from 2 to 13%. [50].

Iron supplements should only be prescribed on medical indication.

### Key points

- Respect the daily caloric intake (70–75 Kcal/day).
- Pay attention to the protein intake (cheese and meat), remembering that two meals of milk (200–300 ml) cover about 30-40% of the daily protein intake.
- Supplement with vitamin D until at least 1 year of age.
- Do not add salt to foods.
- Prefer:
  - starchy foods to simple sugars, reducing the consumption of fruit juices and sweet snacks as much as possible.
  - the consumption of vegetable oils as opposed to animal fats and, especially, olive oil instead of butter and margarine.
  - the consumption of fish (at least twice a week) as opposed to meat.
  - the consumption of fruits and vegetables (4 servings a day, if possible).

## Part IV: Baby Led Weaning and auto-weaning

Baby-Led Weaning (BLW) is a method for introducing complementary foods to infants; in this mode, infants feed themselves with hand-held foods instead of being spoon-fed with purées by parents [53,54].

According to Piermarini L, BLW may also be defined auto-weaning, which means offering chopped and minced family meals to the infants [55,56].

In both methods, infants should be milk-fed, ideally exclusively breastfed on demand and they should be offered complementary foods from 6 months of age.

BLW has many advantages, such as improving relationships during shared family meals, promoting the infant’s autonomy, saving time and money and, perhaps, encouraging healthier dietary intakes for parents. However, a single study [57] has found that BLW does not improve the family’s eating style.

Paucity of data on BLW is a reason for uncertainty and concern for primary care pediatricians.

- *Risk of inadequate iron intake*. It has been found that iron deficiency leads to deficit in cognitive processing [58]. Generally with BLW, families offer vapor cooked vegetables which are not a source of absorbable iron [58].
- *Risk of high NaCl intake*. A high sodium intake in infancy is involved in the development of hypertension in adulthood [46]. An excessive intake of sodium may play a role in autoimmune diseases [46].
- *Risk of insufficient energy intake.* A study comparing different weaning styles found an increased incidence of underweight in a group of 92 baby-led infants and an increased incidence of obesity in a group of 63 spoon-fed infants [42].
- *Risk of choking*. The infant may have not developed the oral motor function required to safely ingest whole foods. In 199 BLW infants, 30% had at least one episode of choking with solid food ingestion (apple). It is possible that this high rate is caused by difficulties of parents in distinguishing choking from gagging [42].

BLW is not recommended before 6 months of age because it is necessary to achieve postural stability to sit and to grasp objects. In practice, most parents adopt a mixed approach to weaning. In only 8% of instances, a strict baby-lead approach is adopted. In most cases, infants had purées foods given by spoon. This permits to ensure iron and caloric intake in some occasions (diseases) (59).

## Final conclusions

- The age of introduction of solid foods should be defined individually, based on the competences acquired and on the interest of the infant towards food (to be assessed together with parents). The beginning of the introduction of solid foods at 6 months of age remains a desirable goal also in Western societies and for exclusively breast-fed infants, although it would be advisable to introduce solid foods together with breast milk before such age. We would however suggest to introduce *complementary foods not before 4 months of age and not after 6 months of age.*
- It is not advisable to delay the introduction of potentially allergenic foods, nor of gluten with the purpose of preventing the development of allergic diseases; there is no ideal timing for gluten introduction in relation with the onset of CD and T1DM.
- It is recommended to encourage the sharing of meal times and the satisfaction of the infant’s curiosity and requests with small tastings of food.
- Offer ground, chopped or finger food only once the child has developed the necessary postural and oral motor skills.
- A *synthesis of BLW and “traditional” solid introduction*, probably spontaneously adopted by many families, allows the child to benefit both from the positive implications of meal sharing and from a nutritionally adequate meal, with attention placed on the moment when *the infant expresses their “desire” to experiment new foods*.
- The child’s diet will be better in as much as the family will follow a *correct and balanced diet*, mindful of the caloric and protein intake. It is therefore of paramount importance to provide parents with the right information about a nutritionally balanced diet, and encourage them to recognize and respect every child’s self- regulatory capacity. It is also important to *promote* the daily *consumption of fruits and vegetables*.
- Solid introduction should privilege family, ethnic and regional habits, considering the nutritional needs of every child.
  *To conclude, it should however be stressed that, based on the literature available on the topic, it is not possible to deduce the necessary number of children for measuring the effect of a certain model of introduction of foods a posteriori, exception made for groups at genetic risk of developing coeliac disease or type 1 diabetes. As soon as more data will be available, such cost-benefit risk analysis will highlight the expected results per population. Such considerations emphasize the urge to be even more cautious in pointing out a model.*
